# Building capacity for medical education research in family medicine: the Program for Innovation in Medical Education (PIME)

**DOI:** 10.1186/s12961-017-0256-y

**Published:** 2017-10-23

**Authors:** Douglas Archibald, William Hogg, Jacques Lemelin, Simone Dahrouge, Mireille St. Jean, François Boucher

**Affiliations:** 1 Department of Family Medicine, University of Ottawa, Ottawa, Canada; 2 Bruyère Research Institute, 43 Bruyère St. Annex E, Ottawa, ON K1N 5C8 Canada

**Keywords:** Health services research, Research design, Faculty, Financing, Organised

## Abstract

**Background:**

Despite the apparent benefits to teaching, many faculty members are reluctant to participate in medical education research (MER) for a variety of reasons. In addition to the further demand on their time, physicians often lack the confidence to initiate MER projects and require more support in the form of funding, structure and guidance. These obstacles have contributed to a decline in physician participation in MER as well as to a perceived decay in its quality. As a countermeasure to encourage physicians to undertake research, the Department of Family Medicine at the University of Ottawa implemented a programme in which physicians receive the funding, coaching and support staff necessary to complete a 2-year research project. The programme is intended primarily for first-time researchers and is meant to serve as a gateway to a research career funded by external grants. Since its inception in 2010, the Program for Innovation in Medical Education (PIME) has supported 16 new clinician investigators across 14 projects.

**Methods:**

We performed a programme evaluation 3 years after the programme launched to assess its utility to participants. This evaluation employed semi-structured interviews with physicians who performed a research project within the programme.

**Results:**

Programme participants stated that their confidence in conducting research had improved and that they felt well supported throughout their project. They appreciated the collaborative nature of the programme and remarked that it had improved their willingness to solicit the expertise of others. Finally, the programme allowed participants to develop in the scholarly role expected by family physicians in Canada.

**Conclusion:**

The PIME may serve as a helpful model for institutions seeking to engage faculty physicians in Medical Education Research and to thereby enhance the teaching received by their medical learners.

## Background

Faculty member involvement in Medical Education Research (MER) is thought to increase adoption of best teaching practices [1, 2]. However, several factors, both personal and environmental, affect a faculty member’s participation in research. Personal factors include confidence, perception and attitude towards research [3]. Faculty members also find it difficult to find the time to conduct research while trying to balance clinical and teaching responsibilities [4, 5], representing a factor that is both personal and environmental because a lack of time is often associated with a lack of leave support. Funding, leadership, mentorship and structure – factors of a supportive environment – have an even greater effect on research productivity than personal motivation, training and time [4–6]. These factors appear to have not only contributed to a global decline of physician-scientists [7], but also to the quality of research being conducted in medical education [8–12]. Quality issues of MER studies are both technical and theoretical. Studies often lack healthcare outcome measurements [13] and control groups, have small sample sizes and employ poor reporting standards [8]. In an evaluation of 390 MER publications, validity and study design scored the lowest of all domains [9]. MER has also been criticised for ignoring relevant literature and for not placing the research question in a wider conceptual context [8].

The most recent Carnegie report, entitled A Call for Reform of Medical School and Residency, recommends creating opportunities for integrating formal learning with clinical experience and also developing habits of inquiry and improvement [1]. The Royal College of Physicians and Surgeons of Canada (RCPSC) has also advocated for contribution to research by physicians, adopting the Scholar role as one of the core competencies of specialty training [14]. The North American Primary Care Research Group (NAPCRG) published guidelines to build research capacity in primary care according to four themes that can be applied to MER, namely building linkages between researchers of varying skill levels and disciplines and between those with a shared topic of interest; providing infrastructure, including research centres with experienced investigators that support each other and mentor others; training, including presenting workshops for research skills dissemination, encouraging and subsidising research-oriented family medicine fellowships; and recognising family medicine researchers for their work, including through grant funding [15].

Determined to build research capacity and to implement new evidence-based educational initiatives within its residency curriculum, the University of Ottawa Department of Family Medicine’s (DFM) research centre the C. T. Lamont Primary Health Care Research Centre (CTLC), launched the Program for Innovation in Medical Education (PIME) in 2010. The present paper provides an overview of this capacity-building programme, which offers faculty development in scholarship and research and improves undergraduate and postgraduate medical education.

### Goals

The goal of the PIME is to promote faculty development and support research and scholarship designed to improve undergraduate and postgraduate medical education in the DFM. In this study, we present two objectives, firstly to determine whether an organised research support programme results in publications of new researchers’ work, and secondly, to determine the effects of a research support programme on participants’ behaviour and the culture of the department. The research question addressed herein is whether an education research support team builds capacity for scholarly work.

## Methods

The PIME was conceived in 2008 by the Chair of the department and his Senior Leadership Team, composed of the Chair, Postgraduate Director, Undergraduate Director and Director of Research. It was launched in 2010 following an extensive design process that included consultations with experts in the field of educational research and members of the university’s Academy for Innovation in Medical Education.

Administered by the CTLC – the research arm of the DFM – the programme is a collaborative initiative between the undergraduate, postgraduate and research portfolios in the department. The Senior Leadership Team determined that sharing infrastructure and resources and building linkages across departments would lead to better research and impactful changes.

### Programme budget

In 2009, the DFM received funding for its teaching and research activities through one of the Academic Health Science Centre’s Alternate Funding Plans in Ontario. The department committed a total of CA$1,125,000 of these funds to the PIME as a mark of commitment to medical education leadership and innovation. This funding covers the salaries of the Education Researcher, the Coordinator and the Research Assistant as well as faculty development and operating costs for a period of 4 years.

### Programme design

For a maximum duration of 2 years, the PIME provides successful applicants with (1) funding and protected time, (2) support and resources, and (3) mentorship by a PhD medical education researcher and the opportunity for collaboration with other researchers. These features have been recommended in the guidelines put forth in the Carnegie report and by the RCPSC and the NAPCRG, described above. The programme is open to all faculty members of the Department of Family Medicine, including non-physicians; however, only family physicians have submitted completed applications thus far.

### Funding and protected time

Investigators receive funds to pay for a half-day locum per week for a period of 2 years (approximately CA$40,000), which provides them protected time to work on the research project. They also received funds of up to CA$7000 for support, including research assistance and statistical consultation.

### Support and resources

The PIME research assistants support investigators with various aspects of the research project, including conducting recruitment, data collection and analysis, and preparing grant applications, manuscripts, abstracts and posters.

The PIME coordinator also provides support by collaborating with the PIME research assistants and coordinating, monitoring and recording the progress of research activities. The PIME coordinator also supports investigators by seeking further funding opportunities and helping them to complete applications. As PIME funding is considered seed funding, investigators are encouraged to apply for additional funding from established sources including programmes within the University of Ottawa such as the Bureau Francophone, the Department of Innovation in Medical Education and Postgraduate Medical Education as well as external programmes provided by organisations such as Physician Services Incorporated Foundation, the Association for Medical Education in Europe and the National Board of Medical Examiners, Edward J. Stemmler Medical Education Research Fund.

Finally, investigators also receive support from statisticians and librarians and have access to workspace and software.

### Collaborative research and mentoring

The PIME promotes collaborative research and mentoring, which has also proven to be effective in improving the quality of MER. PIME is led by a full time, appointed PhD medical education researcher who provides methodological skills that enhance the quality of projects. The researchers spend the majority of time leading their own programmes of medical education research and collaborate with educators in the pursuit of their ideas to improve the quality of teaching and learning of trainees in the DFM. The PhD medical education researcher mentors interested DFM educators as they transform their scholarly and research ideas into feasible projects, offering support to faculty members with research design, data analysis and other research components, and is uniquely positioned to identify and help address any obstacles. If additional methodological expertise is provided, the medical education researcher is added as a co-investigator on PIME-funded projects.

Investigators can consult with scientists in the field of Primary Health Care Research from the Bruyère Research Institute Methods Centre and are encouraged to involve residents in their projects, participate in our mentorship programme and continue to integrate scholarship in their teaching.

### Eligibility

All faculty members of the Department of Family Medicine are invited to submit funding applications to conduct innovative educational research projects once yearly. In establishing priorities among applications submitted, when scientific merit and clinical relevance are equal, preference is given to the new investigator over the established investigator.

### Application process

Submitted project proposals are reviewed in a competitive two-step process involving the submission of the letter of intent and the submission of the full proposal. The letter of intent is first reviewed by the postgraduate or undergraduate family medicine director (based on the focus of the proposed project) and the clinical unit director to ensure that the study aligns with the department’s vision and priorities and that it will not affect clinic resources. Once approved by the directors, the letter of intent is submitted and reviewed by the selection committee. The committee is comprised of expert researchers, clinician teachers, clinician investigators, and medical education researchers from the Department of Family Medicine and the Faculty of Medicine.

Applicants receive feedback and are invited to submit a full proposal based on departmental alignment. During the second step of the process, full proposals are submitted to the selection committee for review and are accepted, accepted with revision or rejected according to established evaluation criteria.

### Selection criteria

In evaluating proposals, the selection committee ensures that the basic principles of high quality MER [11, 16] are followed such as a thorough review of the literature, a good research question and an appropriate sample size and study design to ensure validity. The selection committee specifically evaluates the (1) relevance to family medicine, (2) clarity of objectives, (3) justification of the project, (4) quality of the proposal (design, methodology and analysis), (5) feasibility of timelines and (6) potential impact/dissemination plan.

Reviewers individually rate these criteria on a 5-point Likert scale (ranging from Very Poor to Excellent) and provide comments in a written evaluation form. Reviewers also rate the proposals on a 10-point Likert scale (Not Acceptable to Excellent). Proposals that are rated between 0 and 4 are not funded, those rated 5 or 6 may require resubmission or are not funded, those rated 7 or 8 are funded with minor revisions and those rated 9 or 10 are funded without revisions. Upon individually evaluating the proposals, the committee meets to discuss each proposal and come to a consensus.

### Evaluation of projects

Programme participants are required to submit quarterly progress reports to the PIME coordinator. These reports allow the PIME team to ensure that projects are progressing and to raise any questions and concerns with study methodology or feasibility.

### Evaluation of the programme

In June 2013, an external evaluation of the programme was conducted by the director of faculty development to identify the strengths, limitations and areas of improvement of the PIME. An official letter of exemption from the institute’s Ethics Review Board was obtained before the evaluation began.

The exploratory study was framed by a well-known evaluation framework in health profession education; the modified Kirkpatrick Model [17]. This is because the evaluation sought to understand how an education research support team could build capacity for scholarly work. The evaluation used purposeful sampling to include 14 stakeholders who were invited by the faculty development director to participate in the evaluation; the programme lead, the coordinator, two research assistants, a member of the selection committee, five PIME grant recipients, three clinicians (two physicians and a pharmacist) who participated in stage one but did not submit a full grant application, and a community physician with no direct involvement in the PIME. Interviews were conducted in person or by telephone and audio recorded, transcribed and analysed. The interview transcripts were checked for accuracy by comparing the audio recording to the transcribed text. The interviews were analysed by a researcher and a research assistant (DA and JB) using inductive thematic analysis [18]. Coding of the data was performed using QSR International NVivo software. One member of the research team performed the initial coding for all transcripts. A second coder analysed a sample of the transcripts, and the two coders met to reach consensus on an established list of codes. Once the codes list had been consolidated, the remaining transcripts were re-analysed by the first coder. The first coder then grouped the codes into themes and discussed with the second coder. Data from all participants were pooled and most themes were derived from comments made by various members of the group. One theme was derived primarily from comments made by PIME staff members. The modified Kirkpatrick model was then applied to frame the interpretation of the themes.

## Results

### Themes – Kirkpatrick’s Level 3 – encapsulating participants’ reactions

From the codes, we derived four themes that pertained to Level 1 of Kirkpatrick’s framework – encapsulating participants’ reactions to the PIME.

#### A new challenge

PIME applicants are clinicians who generally do not have experience with MER. Many of the programme participants had never written a grant proposal before, and some expressed that they found the prospect intimidating and that they did not know where to begin. Most of the applicants expressed that the support offered by the PIME relieved the fear of making these first steps in their research career. With the help of the programme, the applicants embraced the grant writing process as a learning experience and a rewarding challenge. Beyond the grant writing process, some participants were intimidated by the research process as a whole, but remarked that the PIME introduces a familiarity with this process. Many participants began the programme thinking that they could not do research and subsequently learned that they can.

“*It takes people who have an interest in research and helps them get started. It is not intimidating and it is not overwhelming*” (Grant recipient)

#### Time and effort

Many of the participants commented that the primary barrier to conducting research is a lack of time; clinical and teaching commitments fill their schedules and take priority over research projects. In anticipation of this barrier, the PIME grants offer the bulk of their funding in the form of protected time – funds paid to the grant recipient’s medical partnership to pay another physician to cover their clinical duties so that the recipient can work on the research project. Nearly all participants, including grant applicants and PIME support staff, indicated this as one of the fundamental strengths of the programme. Unfortunately, for some of the applicants, time constraints proved to be too much of a challenge in completing the grant application.

The lead of the PIME remarked that he was candid with applicants and recipients about the amount of time and effort that would be involved. Once applicants received feedback in response to their letter of intent, they realised how much effort is involved in both preparing the final application and conducting a project and sometimes opted not to continue.

#### Support from PIME

Participants expressed that they felt supported throughout the process by the PIME staff and the programme lead. The programme lead provided extensive coaching and mentoring in the areas of project design and medical education research practices, a contribution that was particularly appreciated by participants.

“*Having* [PIME Lead’s name] *is everything. Having somebody who has that knowledge base and medical education and methodology on what the latest trends are is priceless. He is able to meet with* [grant recipients]*, he is able to install confidence in them, and he is really able to help guide the ideas and he is knowledgeable. It is a good lead for this programme*” (PIME coordinator)

PIME recipients also received administrative support from PIME staff members, including budget management and applications to research ethics boards. Beginning a research project through the PIME unlocks resources and support that empower the grant recipients to take on the challenge.

#### Potential improvements

We received many suggestions for improvements to the programme from applicants and recipients. Though suggested in slightly different formats, many of these involved an organised programme for PIME recipients in which they could regularly present their progress to one another and solicit feedback and discussion. Participants also requested a greater amount of support with writing and dissemination and guidance regarding the next steps once the project is completed.

### Themes – Kirkpatrick’s Level 3 – Changes to one’s behaviour

We interpreted two themes from participants’ responses that related to Level 3 of the Kirkpatrick model – changes to one’s behaviour.

#### Scholarly identity

A number of comments from participants related to the idea that the PIME was making the Department of Family Medicine more scholarly. Participants mentioned that the department was in need of improvement in this regard and, at the time of the interviews, it was undergoing a palpable change. PIME was said to show that medical education research is important to the department, and the rigour of the grant review process lent legitimacy to the department’s research activities. The programme lead remarked that PIME appears to be a unique programme among departments of family medicine across Canada and that it encourages clinicians with compelling research questions to take the first step.

“*The first step is to say is it makes me – and I think a lot of people who are like me and are doing frontline work in the department – see that it is possible to do scholarly work. I think that is how we are going to be a more respected department at the university*” (Grant applicant)

In addition to its effect on the Department, the PIME also appears to have affected the scholarly identity of individual participants. Two grant recipients said that the programme reiterates to clinicians what it means to be scholarly, and one made the connection between participating in PIME and developing in the CanMEDs Scholar role [19]. Similarly, a non-physician applicant expressed that he felt as if he was not a fully realised clinician unless he performed research, thereby contributing to the growing body of knowledge in his field. One participant recognised that her enhanced scholarly approach was improving the very curriculum that her project sought to evaluate. Participation in the PIME appeared to influence individuals’ approach to education and research.

#### Collaboration

Many participants said that their PIME project taught them the importance of having a team, both in the sense of having a research assistant to help with the day-to-day tasks of the project and to have colleagues and co-investigators attached to the project who complement their knowledge and skills. Participating in the programme allowed some participants to form connections with other clinician colleagues who are interested in doing research and also to draw on the different perspectives of non-clinician researchers. Ultimately, this sort of collaboration could create a community of educational researchers within the department.

“*Applying for the grant was a very good learning experience for me. It helped me to understand the whole research process. It was one of those things that made me realize how important it is to have a team with that process*” (Grant recipient)

### Themes – Kirkpatrick’s Level 4 – Results

Finally, we derived two complementary themes that relate to Kirkpatrick’s Level 4 – Results. Interview participants who worked as part of the PIME team outlined the goals of the programme, some of which were also mentioned by grant recipients. Outcomes of individual projects and the programme as a whole arose in interviews for comparison with these goals.

#### Goals of PIME

The most frequently mentioned goal of the programme was for grant recipients to disseminate their research findings through presentations or publications. Participants expressed that a strong dissemination presence would bolster the reputation of the programme and of the department. Such success would secure the longevity of the programme and allow it to grow by attracting new applicants. In order to support more researchers, participants remarked that PIME will need more resources, including more funds. At the time of the interviews, the future funding of the programme was uncertain, and this limited the programme’s capability to make concrete plans. However, some participants expressed that the goal of the programme was to provide seed money and support to start participants’ research careers, with the understanding that they would secure external funding in order to continue their work.

#### Outcomes of PIME

The majority of recipients had given presentations at conferences or had submitted abstracts at the time of their interview. Staff members of the PIME described the number of presentations given by grant recipients as impressive and satisfactory to the programme’s goals. A smaller number of grant recipients had also begun to draft manuscripts. Importantly, many grant recipients had leveraged their early work to apply for additional funding from external sources.

### Outcomes

Receiving four applications in its first year, PIME has maintained the interest of the DFM with 25 letters of interest received from 2010 to 2014. Since its inception in 2010, PIME has funded 14 medical education projects, supporting a total of 16 clinician teachers out of 26 applicants (Table 1). A number of projects that were not initially funded were accepted in the following year as a result of collaborative revision by the PIME team and educators.

**Table 1:** PIME Grant: 2010–2014 summary

Indicators	Total
Project funding and support	
Projects funded	14
Projects completed	10
Supported clinician teachers	16
Dissemination	
Publications to date (in press or submitted)	–
Published	6
Conference presentations to date (posters, workshops, oral)	30
External funding	
External funders (including two summer studentships)	4
Total external funding	CA$61,646

Whether evaluating a website that creates learning plans for residents by educators or seeking to improve the teaching and learning of interprofessional collaborative practice for medical students and residents (Table 2), all funded projects have sought to advance family medicine education through research and innovation.

**Table 2:** Funded projects

Project title	Number of Department of Family Medicine faculty investigators	Year funded	Current status/next steps
The academic support process website: evaluation of an online tool designed to support preceptors	2 co-principal investigators; 1 co-investigator	2010	Project successfully completed Published manuscripts [20, 21]
Interprofessional education placement for students on the palliative care unit	2 co-principal investigators	2010	Project successfully completed
Improving family medicine residents’ commitment to practicing intra-partum obstetrics after graduation	2 co-principal investigators	2010	Project successfully completed
Assessing the impact of a care of the elderly rotation on geriatric learning in family medicine residents	2 co-principal investigators	2011	Project successfully completed
Evaluation of a humanities curriculum in a family medicine teaching programme	1 principal investigator; 2 co-investigators	2011	Project successfully completed
Does the global and refugee health e-learning programme increase medical students’ conceptual knowledge of global health?	1 principal investigator; 1 co-investigator	2012	Project successfully completed Published manuscript: [22]
Developing and using a resident practice profile to enhance learning in family medicine residency	1 principal investigator; 1 co-investigator	2012	Project successfully completed Published manuscripts: [23, 24]
In-training practice Simulated Office Orals (SOOs) vs. College of Family Physicians of Canada’s (CFPC’s) Certification Examination Scores: a correlation study	1 principal investigator	2012	Project successfully completed Published manuscript: [25]
The effects of a mindfulness curriculum in undergraduate medical education on wellness-related outcomes	2 co-principal investigators; 1 co-investigator	2013	Project successfully completed Manuscript writing in progress
Does a structured curriculum and procedure clinic help family medicine residents diagnose and treat skin cancer?	1 principal investigator	2013	Project successfully completed Manuscript writing in progress
What are the affordance and constraints of professional identity formation for palliative care in family medicine?	1 principal investigator	2014	Project successfully completed
Development and evaluation of a program to introduce humanities to teachers of clinical medicine	1 principal investigator	2014	Project successfully completed Manuscript writing in progress
Attitudes toward professionalism in medical education	1 principal investigator	2014	Project successfully completed Published manuscript: [26]
Building and modelling skills for comprehensive and integrated women’s healthcare in family medicine: moving pessary care from a family medicine focused clinic to routine primary care in an academic family medicine practice	1 principal investigator	2015	Data analysis in progress

To date, recipients have presented 30 conference presentations and published six articles in peer-reviewed journals with more publications in process (Tables 1 and 2). Four recipients have successfully secured additional external funding following PIME funding (Table 1).

## Discussion

Though the outcomes demonstrate a modest return on investment, these results are preliminary, particularly considering that many of the projects listed were only recently completed. Cultural change requires time and it appears that, in the short term, outcomes are positive as outlined by all stakeholders surveyed during the evaluation.

Fitting interview responses into the Kirkpatrick framework requires us to acknowledge that we did not address Kirkpatrick’s Level 2 – the change to participants’ knowledge following participation in our programme. The study did not include a measure for knowledge attained, in part because the technical details of each project were different. A future study could evaluate participants’ understanding of general research principles including the writing of grant proposals following their participation in PIME.

Kirkpatrick’s Level 1 – Reaction, was addressed by four of our themes. There is a degree of ambivalence among the themes at this level, namely, participants found the programme to be very challenging and that it required a lot of time and effort, but also felt amply supported throughout the process. Ultimately, this suggests that their participation was an educational and valuable experience that required a great deal of growth.

The third level of Kirkpatrick’s model – Behaviour, can be difficult to assess, particularly when using self-reported data. In our study, participants described an ongoing cultural shift within their department toward a greater presence of scholarly conduct, which they suggested may have been brought about by PIME. As indicated by some participants, this shift can bolster the reputation of the department within the university, a benefit that could be leveraged by other departments. A new appreciation for collaboration also emerged among most of the participants, due in part to the nature of the challenge – as many of the recipients were new to research or specific kinds of research, they discovered the need to form a team and learned to reach out to colleagues and non-physician researchers for support.

The themes pertaining to Kirkpatrick’s Level 4 – Results, were complementary to one another. The goals of PIME that were articulated primarily by PIME staff included dissemination of research results and applications for external funding. Many participants had disseminated their work through conference presentations or publications and some had secured external funding for their work. The ability for PIME alumni to secure external funding may become very important for the longevity of the programme if the internal funding becomes unstable.

The themes that we interpreted from interview data suggest that the programme precipitated a positive cultural change in the department. Programmes that provide protected time for research may be successful in other contexts to foster the development of a scholarly environment.

### Lessons learned

Sustainability may be viewed as a limitation of the PIME initiative. Initial funding was limited to a 4-year plan. Since 2014, funding has been renewed on a yearly basis and according to the number of applications received. In order to continue the work being done, funding must be stable.

The scope of the programme was initially limited to MER and scholarship. In 2015, a proposal to allow the PIME to expand and support all primary care-related research was submitted and accepted. This research was previously supported by a second internal funding programme which has since been abolished. Having one funding programme with a clear approach was viewed as advantageous. The programme is now the called the Program for Research and Innovation in Primary Care and Medical Education and receives funding on a yearly basis.

During consultations with various stakeholders, the CTLC discovered that protected time was not viewed as the best use of resources and has redirected funding toward support personnel and services.

Dissemination of research findings thus far could be improved. In recent years, we have been actively encouraging PIME recipients to submit abstracts to conferences; this has yielded greater engagement by the recipients in the form of oral presentations and posters. Several investigators have completed data collection within the past year and are preparing manuscripts for submission to medical education journals.

### Next steps

Since 2015, an additional three projects have been funded. The expanded scope of the programme promises exciting research in both Medical Education and Primary Care research. The Department of Family Medicine has committed to supporting operation funds for up to four research projects per year in the amount of CA$20,000 each.

Individual programme participants have secured additional funding through other granting programmes within the faculty and provincial grants. Present and future grant recipients will be encouraged to apply to these granting organisations and others to maintain and expand their own programmes of research.

### Limitations

This study represents an evaluation of a granting programme within one family medicine department at one university. The small number of interview participants and grant recipients limits the scope of conclusions that can be drawn from this work. Some interviews were performed in person while others were performed by telephone; this meant that non-verbal nuance is lost in the telephone interviews but was necessary to accommodate some of the participants. Not all grant recipients or applicants participated in this evaluation and thus important perspectives may have been inaccessible.

## Conclusions

In accordance with recommendations of respected organisations such as the Carnegie Foundation, NAPCRG and the RCPSC, the PIME has attempted to build research capacity to ultimately increase the adoption of best teaching practices by targeting the personal and environmental factors that deter faculty members from engaging in and producing high quality MER. The PIME offers faculty members time and funding to pursue their research interests and, more importantly, a supportive infrastructure, mentorship and opportunities for collaborative research.

While it may be too early to report the impact of PIME on teaching practices and undergraduate and postgraduate education based on outputs, outcomes highlight a much needed culture change within the department. By facilitating the efforts of family medicine educators and ensuring the rigorous conduct and dissemination of their innovations, trainees at the University of Ottawa and elsewhere will acquire the knowledge and skills to serve the needs of patients and community.

## Abbreviations

CTLC: Campbell T. Lamont Primary Health Care Research Centre
DFM: Department of Family Medicine (at the University of Ottawa)
MER: Medical Education Research
NAPCRG: North American Primary Care Research Group
PIME: Program for Innovation in Medical Education
RCPSC: Royal College of Physicians and Surgeons of Canada
